# No evidence for a preferential role of sleep in episodic memory abstraction

**DOI:** 10.3389/fnins.2022.871188

**Published:** 2022-12-09

**Authors:** Lucia M. Talamini, Dirk van Moorselaar, Richard Bakker, Máté Bulath, Steffie Szegedi, Mohammadamin Sinichi, Marieke De Boer

**Affiliations:** ^1^Brain and Cognition, Department of Psychology, University of Amsterdam, Amsterdam, Netherlands; ^2^University of Amsterdam—Amsterdam Brain and Cognition, Amsterdam, Netherlands

**Keywords:** episodic memory, regularity extraction, generalisation, sleep, memory consolidation

## Abstract

Substantial evidence suggests that sleep has a role in declarative memory consolidation. An influential notion holds that such sleep-related memory consolidation is associated with a process of abstraction. The neural underpinnings of this putative process are thought to involve a hippocampo-neocortical dialogue. Specifically, the idea is that, during sleep, the statistical contingencies across episodes are re-coded to a less hippocampus-dependent format, while at the same time losing configural information. Two previous studies from our lab, however, failed to show a preferential role of sleep in either episodic memory decontextualisation or the formation of abstract knowledge across episodic exemplars. Rather these processes occurred over sleep and wake time alike. Here, we present two experiments that replicate and extend these previous studies and exclude some alternative interpretations. The combined data show that sleep has no preferential function in this respect. Rather, hippocampus-dependent memories are generalised to an equal extent across both wake and sleep time. The one point on which sleep outperforms wake is actually the preservation of episodic detail of memories stored prior to sleep.

## Introduction

Most current accounts of long-term memory incorporate the notion of interacting, complementary memory stores: a highly plastic store that quickly encodes the unique conjunctions of different event components, and a less plastic one that gradually discovers the structure across experiences (McClelland et al., 1995; Martin and Chao, 2001; Frankland et al., 2004; Meeter and Murre, 2005; Moscovitch et al., 2005; Binder et al., 2009; Winocur et al., 2010; Battaglia and Pennartz, 2011; Rasch and Born, 2013). The first store, attributed to the hippocampus, crucially underlies episodic memory function, while the second, corresponding to widespread (neo)cortical regions, stores higher-order general knowledge, akin to the concept of semantic memory. According to this notion, the formation of general knowledge, in particular knowledge about higher-order, associative regularities, depends on a hippocampo-neocortical dialogue, in which the hippocampus serves as a buffer, storing higher-order input configurations sufficiently long to allow build-up of extrahippocampal neural representations reflecting the regularities across temporally and spatially discontinuous inputs (Gordon Hayman et al., 1993; Rosenbaum et al., 2001; Manns et al., 2003; Bayley and Squire, 2005; Moscovitch et al., 2005; Sweegers et al., 2014). Thus, on one hand, memories for statistical regularities, which are important for predicting behaviour in the long run, would acquire a neural representation that is relatively resistant to decay. On the other hand, arbitrary conjunctions would tend to be forgotten at a relatively high rate, consequent to fast overwriting of neural patterns in the hippocampus (Talamini and Gorree, 2012).

From the early days of memory modelling, it has been hypothesised that the above-mentioned hippocampocortical consolidation process, sometimes referred to as system-level consolidation, is facilitated by sleep (Crick and Mitchison, 1983; Alvarez and Squire, 1994; McClelland et al., 1995; Squire and Alvarez, 1995; Robins, 1996; Robins and McCallum, 1999; Meeter and Murre, 2004; Diekelmann and Born, 2010; Lewis and Durrant, 2011; Klinzing et al., 2019). In support of this notion, experiments in rodents have shown that neuronal firing patterns resembling day-time patterns occur during sleep (Pavlides and Winson, 1989; Skaggs and McNaughton, 1996). This so-called “replay” is initiated in the hippocampal CA3 area, in the form of sharp-wave ripples, and propagates to the entorhinal cortex and beyond to recruit activity in neocortical and other extrahippocampal areas. Sharp-wave ripples are temporally coupled to thalamocortical sleep spindles, reflecting memory reactivation at the cortical level (Cox et al., 2014a), and with slow oscillations, which are thought to play a role in the spatiotemporal coordination of memory reactivation (Cox et al., 2014c). The occurrence of each of these sleep oscillations has been linked to memory consolidation (Eschenko et al., 2008; Girardeau et al., 2009; Mednick et al., 2013; Ngo et al., 2013; Fogel et al., 2017). Furthermore, a large number of studies have shown that sleep benefits learning and memory consolidation in humans (Walker and Stickgold, 2004; Gais et al., 2006; Rasch and Born, 2013; Alger et al., 2015; Feld and Diekelmann, 2015). The combined findings provide convincing support for the role of sleep in memory reactivation and consolidation.

Interestingly, however, a large majority of studies on episodic memory consolidation have assessed the role of sleep in strengthening or stabilising memories in their studied form (Walker and Stickgold, 2004; Gais et al., 2006; Rasch and Born, 2013; Alger et al., 2015; Feld and Diekelmann, 2015), testing, for instance, how sleep or wake after learning influenced the retention of studied items and their sensitivity to interference (Ellenbogen et al., 2006, 2009; Talamini et al., 2008; Diekelmann and Born, 2010). While some studies did address the role of sleep in the discovery of rules and regularities (Gómez et al., 2006; Ellenbogen et al., 2007; Tamminen et al., 2012; Durrant et al., 2013), most of these used tasks in which memory domains besides episodic memory, including procedural and semantic memory, likely played a role. As such, the encoding of the individual items is not crucially dependent on hippocampal function, and any regularity extraction is not necessarily dependent on a hippocampocortical dialogue.

Thus, while notions on the role of sleep in hippocampo-cortical recoding and concomitant memory abstraction have been highly influential (Crick and Mitchison, 1983; Alvarez and Squire, 1994; McClelland et al., 1995; Squire and Alvarez, 1995; Robins, 1996; Robins and McCallum, 1999; Meeter and Murre, 2004; Walker and Stickgold, 2004, 2010; Diekelmann and Born, 2010; Winocur et al., 2010; Battaglia and Pennartz, 2011; Lewis and Durrant, 2011; Nadel et al., 2012; Rasch and Born, 2013; Dudai et al., 2015), support from experimental findings is scarce. Indeed, only a few studies address this directly (Cox et al., 2014b; Sweegers and Talamini, 2014; Sweegers et al., 2014, 2015). The pertaining studies, from our lab, used memory tasks that were carefully designed, such that the encoding of exemplars is crucially dependent on the hippocampus. In accordance with the extensive literature on the necessary role of the hippocampus in conjunctive coding (Graham and Hodges, 1997; Eichenbaum, 2000; O'Reilly and Rudy, 2001; Ergorul and Eichenbaum, 2006), this was ensured by using tasks requiring the fast encoding of many new associations between non-semantically related, higher-order constructs. Importantly, the information to be associated is represented in different cortical areas, to minimise any contributions from local processing to performance. Typically, we use arbitrary pairings of faces or objects (represented in fusiform area) with egocentrically processed locations (represented in posterior parietal areas). Three previous fMRI studies confirm that, indeed, these types of tasks recruit the hippocampus, as well as the representational areas of faces or objects and locations (Giovanello et al., 2003; Zeineh et al., 2003; Takashima et al., 2007, 2009; Staresina and Davachi, 2009; Westerberg et al., 2012; Sweegers et al., 2014). Finally, regularities in the presented material are only present, and can thus only be extracted, over hippocampus-encoded, conjunctive aspects of the items. Moreover, the regularity structure embedded in the task material is sufficiently complex to ensure slow and gradual development of regularity knowledge, across many exemplars. This complexity also ensures that understanding of the regularity structure remains partial in all participants, allowing for further offline development after the learning phase.

The results of our earlier studies on generalisation across hippocampus-dependent memories (i.e., new exemplars; Sweegers and Talamini, 2014; Sweegers et al., 2014) indicated that regularity knowledge can be acquired across episodic exemplars and generalised to new situations featuring new exemplars. The build-up of this knowledge was shown to involve the coordinated activity of the hippocampus and mediofrontal regions (Sweegers et al., 2014). It was, furthermore, found that regularity extraction hampered the storage of arbitrary episodic features, resulting in an impoverished memory trace (Sweegers and Talamini, 2014). Finally, across a period of several weeks, memory for the regularity structure appeared very robust, whereas memory for arbitrary associations showed steep forgetting (Sweegers and Talamini, 2014).

Two of our studies addressed the role of sleep in generalisation across episodes (Cox et al., 2014b; Sweegers and Talamini, 2014); one of these assessed the benefit of context cues on retrieval across 12 and 24-h intervals beginning with either wake or sleep, expecting that, as memories generalise and lose spatiotemporal context information, these benefits should diminish. It was found that whereas contextual cues lost their potency with time, sleep did not modulate this process (Cox et al., 2014b). The other study focused on the development of regularity knowledge over episodic exemplars across a 4-h period containing a 2-h nap or no nap (Sweegers and Talamini, 2014). While generalisation performance increased across the post-learning interval, this occurred to a similar extent for the sleep and wake condition. The combined findings corroborate several predictions of complementary learning systems models, including the involvement of a hippocampo-cortical dialogue in episodic regularity extraction, preferential occurrence of system-level reorganisation for associative regularities, and temporal stability of consolidated regularities vs. fast decay of arbitrary episodic details. However, the putative role of sleep in these processes is contested.

A limitation of the study on the role of sleep on regularity extraction (Sweegers and Talamini, 2014) concerns the brief sleep opportunity, which may not allow the beneficial effects to sufficiently develop. We, therefore, present two novel studies, on independent subject samples, which compare generalisation across episodes over longer consolidation intervals. The two studies capitalise on different aspects of the generalisation process and adopt differential sleep–wake interventions (comparison of natural sleep and wake intervals vs. sleep deprivation). As such the likelihood of alternative interpretations for our results is reduced. Both studies assess cross-episodic regularity extraction using tasks that require the learning of a large set of face-location associations and later retrieval of locations based on face cues. As in the study by Sweegers and Talamini (2014), the face-location item sets feature complex regularities regarding the combinations of facial features and locations. Synthetically constructed faces allow us to systematically manipulate and fully control the presence of face-location regularities. Importantly, the tasks are designed such that the presence of regularities is not obvious and regularities can only be extracted across many face-location items. This means that the build-up of hippocampus-dependent representations of individual face-location associations is a necessary step towards regularity extraction.

**Figure 1:**
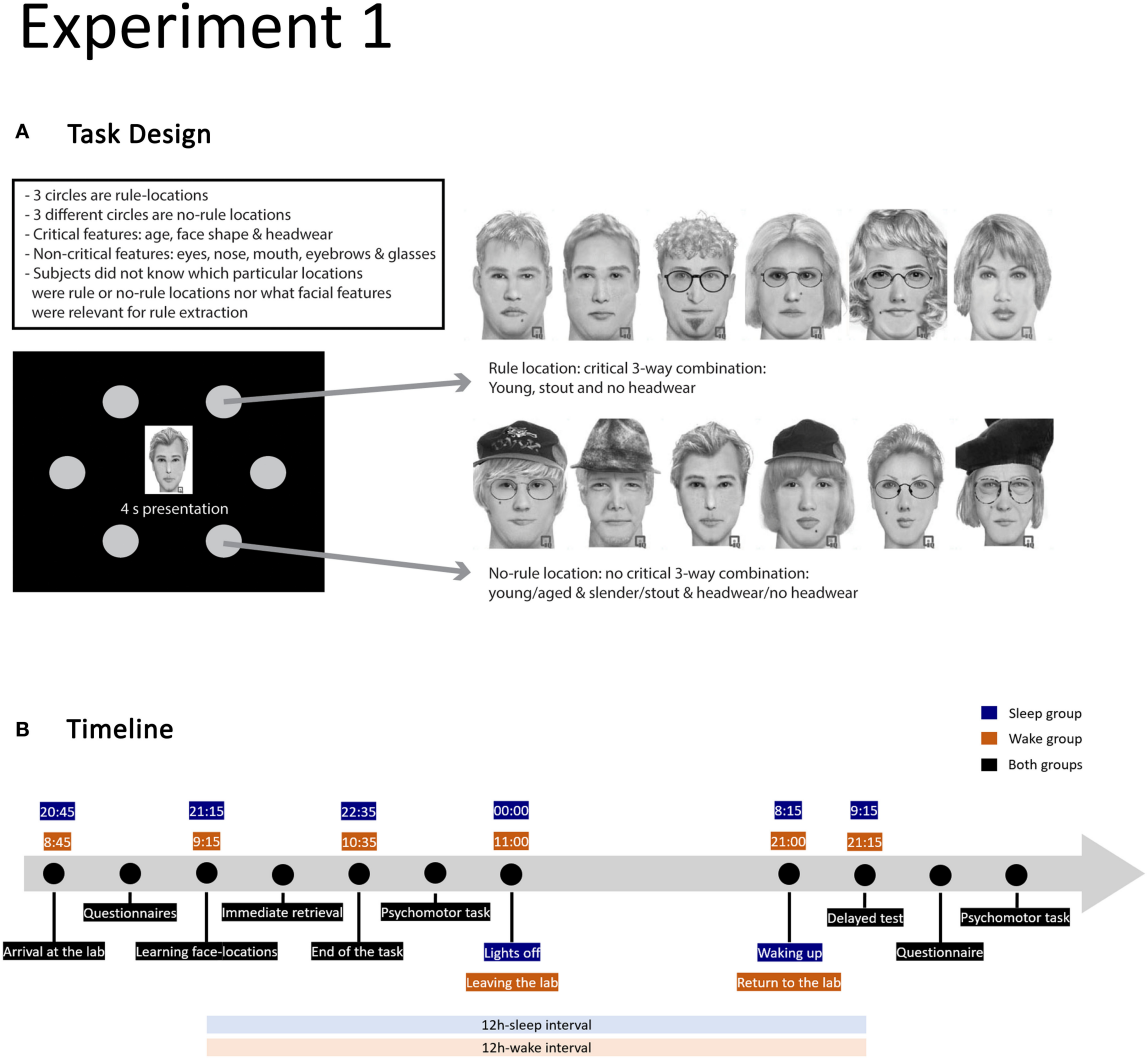
Design of experiment 1. **(A)** Illustration of the task design with examples of faces for one rule and one no-rule location (adopted from Sweegers et al., 2014). The task features 3 rule and 3 no-rule locations with 12 faces associated with each location. The faces corresponding to a rule-location have a unique combination of three features (age, face shape, and headwear); each rule location features a different unique combination. For faces associated with the no-rule locations, the occurrence of critical features and their combinations is random. **(B)** Timeline of the experimental procedure in the sleep and wake conditions.

Experiment 1 compares retention of regularities over a 12-h period containing a full night of sleep or day-time wakefulness. Subjects are trained on a face-location memory task in which half of the items respond to regularities, whereas the other half does not (Figure 1). They are told that some items respond to regularities that may help them to perform the task. Shortly following acquisition, and after a 12-h delay containing either sleep or waking, face-cued location retrieval was tested. More importantly, at these same times, we also assessed generalisation of extracted regularity knowledge to new face-location exemplars, as well as memory for an arbitrary (regularity-irrelevant) episodic memory component: the temporal order in which face-location items were shown during training. With the latter part of the task, we aim to extend our findings regarding the effects of generalisation on encoding and retention of arbitrary details. Indeed, in our previous experiment, these details were arbitrary facial features, which can in principle be learned independently of the hippocampus. In the current experiment, the arbitrary feature, temporal order, is a hippocampus-dependent contextual feature (Dusek and Eichenbaum, 1997; Kesner et al., 2002; Ergorul and Eichenbaum, 2006).

**Figure 2:**
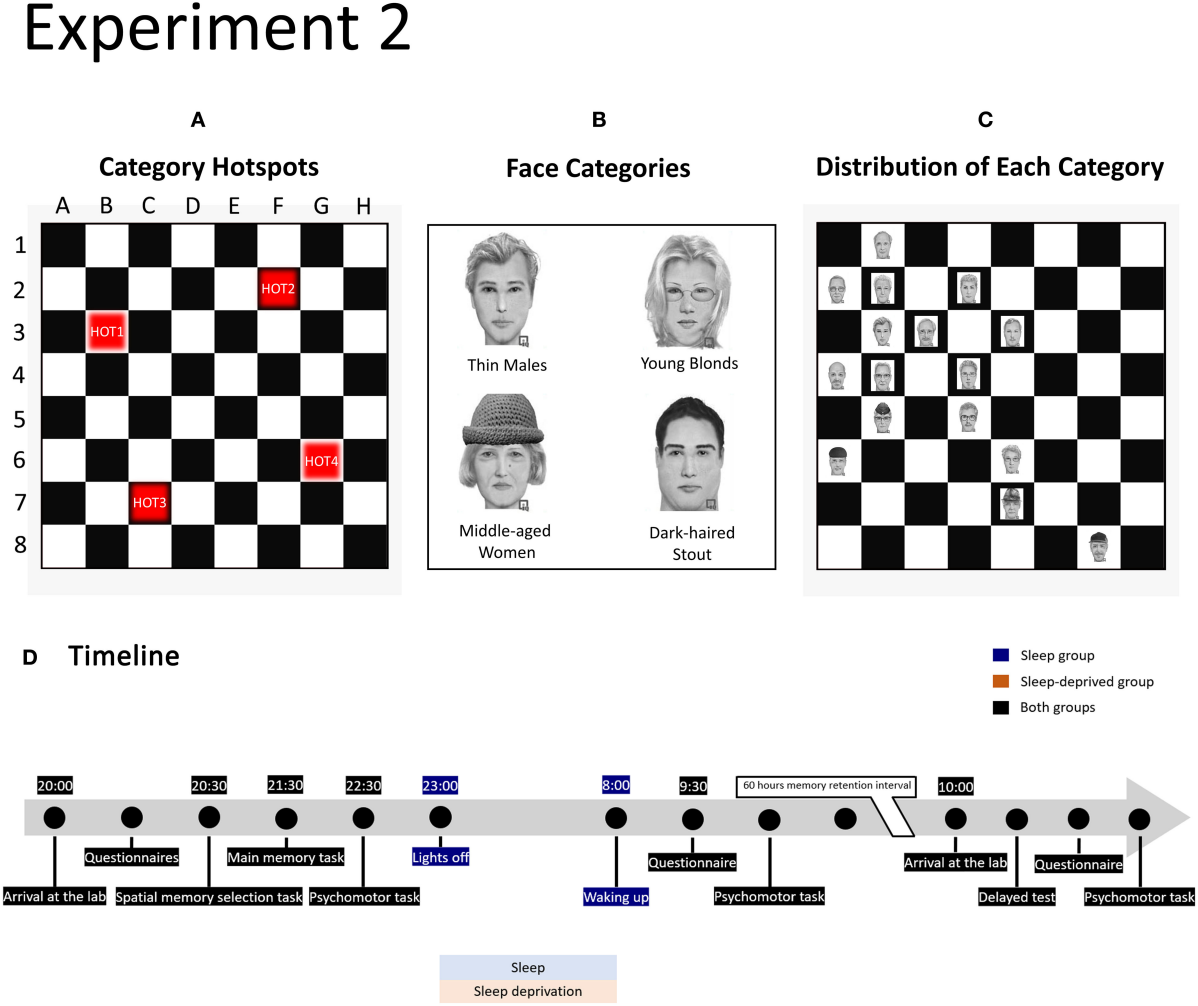
Design of experiment 2. **(A)** Position of category hotspots on the memory board. **(B)** Example for each of the four face categories. Each category holds 16 different faces featuring a combination of two features that is unique to that category. The four face categories are each coupled to a category hotspot (coupling was randomised across participants). **(C)** Example of the distribution of faces in one category (here Thin Males) around hotspot 1. The hotspot location always features a correct category face. For locations, one square removed from the hotspot, 5 of the 8 squares hold correct category faces (62.5%) and 3 squares hold other face categories (37.5%), and so forth. **(D)** Timeline of the experimental procedure in the sleep and sleep-deprived conditions.

Experiment 2 capitalises on implicit generality extraction. To this purpose, a task was developed that requires subjects to learn a large set of face-location associations in a chessboard-like field (Figure 2). Unbeknownst to subjects, each face belonged to one of four categories, defined according to combinations of specific facial features. Each category of faces had its own “hot spot” in the field. The spatial distribution of a set of category faces around this hot spot was approximately Gaussian, with the maximum density over the hot spot (Figure 2). Subjects were instructed to remember the location on the chessboard of each face. Memory for the face locations was tested after a 60-h retention interval, containing either a normal sleep–wake rhythm or a first-night sleep deprivation followed by two recovery nights. A memory accuracy measure was defined as the average spatial error over all retrieved face locations. A generalisation measure was defined as the average deviation of spatial errors in the direction of the hot spots. This reflects the underlying assumption that the build-up of general knowledge regarding the distribution of specific face types in space contributes to retrieval performance and drives responses in the direction of the hot spots.

The combined evidence across our current and previous experiments should provide definitive evidence to either support or dispute the sleep generalisation hypothesis.

## Methods

### Stimuli

For both experiments, computer-generated, grey-scale pictures of emotionally neutral faces were created using Faces Edu Plus (IQ Biometrix, 200). Each face had a unique appearance but contained critical facial features that were systematically manipulated. These “binary” features could take on one of two values. The combination of critical features defined face categories.

Experiment 1, involved three critical features: (1) age (young adult or middle-aged) (2) facial shape (stout or slender) (3) presence or absence of headwear (caps, hats, or headbands). For 6 out of the 8 possible combinations of these 3 features, 24 faces were created, resulting in 144 faces in total. Each of the six face categories had one unique combination of two features (e.g., thin and no head accessory) that did not occur in other categories. We divided the resulting 144 faces into 2 sets of 72 faces in total. The first 72 of these were used in the spatial task with 12 faces for each of the 6 locations. The second set of 72 faces was further divided into two sets of 36 faces that were used in the two generalisation tasks (see in next section).

In experiment 2, four binary features were systematically manipulated: age (young adult or middle-aged), face shape (slender or stout), hair colour (dark or blond), and gender (female or male). A four-way combination of these features led to 16 face categories, each with a unique combination of two features that did not occur in other categories. Four of these categories were used in the present experiment: thin males, middle-aged women, young blonds, and dark-haired stout faces. For each category, 16 faces were created. Faces were presented on a memory board, comparable to a chessboard, which consisted of 64 black and white squares (8 × 8). The board was presented on a grey background.

Importantly, because of the way face stimuli are created, all critical facial features and all possible combinations of critical features are equally frequent in the total pool of faces presented during one task. The only regularities in the tasks are, therefore, defined as feature(s) to location associations, requiring the hippocampus to be encoded. Stimuli were presented with a Presentation (version 9.7, NeuroBehavioral Systems, Inc.).

### Sleep quality measures and vigilance task

The Stanford Sleepiness Scale (SSS) was used to assess momentary sleepiness (Hoddes et al., 1973), the Sleep Quality Scale (SQS) to measure sleep quality over the previous night (Yi et al., 2006), and the Pittsburgh Sleep Quality Index (PSQI) to assess sleep quality and disturbances over the last 1-month time interval (Buysse et al., 1988). A Psychomotor Vigilance Task (PVT; PEBL platform) was used to measure sustained attention and detect sleep lapses (Wilkinson and Houghton, 1982). In this task, subjects were asked to press a button as soon as possible when a red circle appeared on the screen. The light turned on at variable intervals, every few seconds. For the vigilance task, average reaction times (RTs) were calculated, as well as attention lapses, defined as responses with an RT exceeding 500 ms.

### Experiment 1 (episodic regularity extraction across natural sleep and wake episodes)

#### Participants

Forty university students gave written informed consent and received either course credits or financial compensation for their participation. Sleep problems (self-perceived or diagnosed), irregular sleep schedules (habitual sleep pattern with <7 h sleep or sleep outside the window 11 p.m.−10 a.m.), habitual day-time napping, (history of) neurological or psychiatric disorders, and use of medication led to exclusion from the experiment. Participants were asked not to consume coffee, alcohol, or any other kind of drug from 24 h prior to testing. Participants had also been informed prior to appearance in the lab that a minimum score of 40 (55.56%) was required in the first spatial memory test; failing to reach this score ended participation. Three subjects were excluded because they did not reach this minimum required score. Analyses were, therefore, performed on 37 subjects (mean age = 22.25, 24 females). The experiment was approved by the local ethics committee.

#### Face-location generalisation task

The face-location generalisation task (adapted from Sweegers and Talamini, 2014) aims to assess the extraction of regularities across face-location items, while at the same time, testing how regularity extraction may affect the storage of arbitrary, regularity-irrelevant episodic features. Accordingly, the learning session is followed by three types of memory assessment: (1) face-cued location retrieval, (2) face-cued temporal order memory (regularity-irrelevant episodic feature), and (3) generalisation assessed through the placement of new category face exemplars. Extensive task development and pilotting served to balance performance level across the three tasks. Herein, we capitalised on achieving an optimal level of performance (i.e., well above chance level, but well away from the ceiling) on the generalisation and temporal order tasks, which test the primary and secondary research question, respectively. Consequently, it was accepted that performance on face-cued location retrieval, which relies on a combination of memory for individual face-location associations (episodic memory) and regularity knowledge, is on the high side.

The task required participants to learn the coupling of 72 faces to 6 screen locations, organised hexagonally around a mid-screen fixation cross. Participants were also asked to remember the order in which the faces were shown. Half of the face-location items responded to regularities that could be gradually extracted across learning rounds (Figure 1). Indeed, three locations were “rule-locations”, meaning that all 12 faces associated with that location belonged to the same category; face categories were defined by three facial features and each rule location was associated with a different facial category, making the regularity structure in the material highly complex and virtually impossible to discover fully during the training session. The other three locations were “no-rule-locations”, and the faces in the three remaining categories were randomly assigned to these locations. The position of the rule/no-rule locations, as well as the assignment of face categories to (rule and no-rule) locations, was counterbalanced over subjects.

The learning phase consisted of four encoding–retrieval cycles (a short break was given after each cycle). During an encoding block, each of the 72 faces popped up over the mid-screen fixation cross and moved to one of the six locations. The order of faces was fixed for each subject, but random across subjects. Immediately after each encoding block, a retrieval block followed in which faces appeared in random order over the fixation cross. Subjects were instructed to indicate the correct location of each face, using a joystick. In the first three cycles, subjects received feedback on each placement: if the correct location was chosen, a green circle appeared at the correct location, and the face moved to that location; if an incorrect location was chosen, a red circle appeared at the incorrect location, followed by a green circle at the correct location. Subsequently, the subject had to make a movement to the correct location, after which the face moved to that location.

After the fourth encoding block, subjects performed a 5-min counting-back task, to avoid contributions of working memory to performance on the subsequent memory test, which assessed face-location memory and, critically, temporal order memory and generalisation of regularity knowledge to new items. During these memory tests, no feedback on performance was given.

The memory test started with the assessment of spatial and order memory. The 72 faces from the learning phase appeared sequentially in random order. Subjects performed two sub-tasks with each face. First, the face appeared above a horizontal timeline with 72 slots marking the temporal order of face presentations. Subjects were asked to indicate when they thought the face had been presented by placing it in a slot on the timeline (forced-choice; self-paced). Next, the hexagonal place field was shown; the face reappeared in its centre for 2 s. Subjects were instructed to indicate the location associated with the face (forced-choice; self-paced). Once they had chosen a location, subjects rated their response confidence on a five-point scale (1 = guessing to 5 = absolutely sure).

Next, subjects went on to the Generalisation task, in which they were asked to place 36 novel faces, each presented in the middle of the hexagonal place field, on a location where it might belong (forced-choice, 4 s response time). Among the new faces, 18 belonged to the categories of the three rule locations and served to assess generalisation of regularity knowledge to new exemplars; 18 new no-rule faces, for which no “correct location” existed, were included to maintain an equal occurrence of all facial features across the set but were not used to assess generalisation. Each response was again followed by a confidence rating on a 5-point scale.

The scores on these tasks served as the baseline measures of spatial and temporal memory, and level of generalisation. These same memory tasks were repeated after 12 h, with a new set of 36 faces for the generalisation task (see Section Procedure).

#### Procedure

Participants in the experiment followed normal sleep–wake cycles and were randomly assigned to one of two retention interval conditions: (1) 12 h-wake (*N* = 18), (2) 12 h-sleep (*N* = 19).

Subjects in the wake group arrived at the UvA-sleep lab at 08:45 in the morning, while those in the sleep group arrived at 20:45. They signed an informed consent statement, filled out the Stanford Sleepiness Scale (Hoddes et al., 1973) the Sleep Quality Scale (Yi et al., 2006), and a demographic data questionnaire, and then started the learning procedure of the Face-location generalisation task. The instructions were to learn as many face-location associations as possible and to memorise the order in which faces were presented during the passive (i.e., without retrieval tests to enhance encoding) encoding blocks. Furthermore, subjects were instructed that some of the items responded to regularities that could help them place the faces. The learning and immediate retrieval session took from 9.00 till ± 10.35 (wake group) or 21.00 till ± 22.35 (sleep group). The wake group subjects then left the lab and came back for the delayed test the same evening. The sleep group subjects prepared for bed and slept in the sleep lab (no physiological signals recorded). The lights were turned off at around midnight. After waking up at 8.15, subjects had an hour to get ready for the delayed test.

Subjects performed a 20-min psychomotor vigilance task (Mueller and Piper, 2014), after both the immediate and the delayed test.

#### Data analysis

Memory for face locations was expressed as the percentage of correctly retrieved locations. Temporal order memory was expressed in terms of temporal error, i.e., the nr of positions away from the correct position, averaged across all retrieved locations in a retrieval session. Generalisation was expressed as the percentage of new face exemplars from rule face categories correctly allocated to the corresponding screen location.

Following tests for normality and homogeneity of variance, generalisation responses were square root transformed to normalise the data. All variables were then analysed with parametric tests.

### Experiment 2 (effects of sleep deprivation on implicit regularity extraction)

#### Participants

Fifty participants were recruited. Exclusion criteria of experiment 1 also held for experiment 2. In view of the impact of sleep deprivation, additional exclusion criteria were current cardiovascular disorders or complaints, current diabetes, any other current disease or disorder that might negatively influence the participants' fitness, and, finally, particular sensitivity to the effects of sleep deprivation. As in experiment 1, participants were asked not to consume coffee, alcohol, or any other kind of drug from 24 h prior to testing.

The local ethics committee approved the experiment, and all participants gave written informed consent. Participants received course credits or monetary compensation for their participation. Three participants did not complete the study, because their score on a spatial memory selection task was insufficient. One participant abandoned the experiment for personal reasons. Analyses were conducted on the data of the 46 remaining participants (33 females; mean: 21.40; SD: 2.24).

#### Spatial memory chessboard task

In this task, participants were required to learn the one-to-one coupling of 64 faces to 64 locations on an 8 by 8 chessboard. Unbeknownst to subjects, there were 4 face categories (thin males, middle-aged women, young blonds, and dark-haired stout faces), each of which occurred preferentially in a particular area of the board. Specifically, the 2D spatial distribution of each category of faces in the field was approximately Gaussian, with the maximum density over a particular “hot spot” (Figure 2). The coordinates of the four hot spot locations were B3, F2, C7, and G6 (Figure 2). The coupling between hot spot locations and face categories was randomised over participants. We programmed two scenarios with similar, but slightly different, distributions of category faces on the memory board. Participants were semi-randomly assigned to scenario 1 or scenario 2.

During encoding, each of the 64 faces appeared sequentially, in the appropriate chessboard square, was gradually enlarged (to optimise visibility) and shrunken again to the size of the square (5 s total). The full set of face-location pairings was presented three times consecutively, in a fixed order.

After a 60-h retention interval, participants were again presented with all 64 faces, which now appeared, one by one, on the right side of the memory board. Participants were asked to place the faces in their original position using arrow buttons on the keyboard; choices were forced and self-paced. After each response, participants were asked to rate their response confidence on a 5-point Likert scale (1 = guessing, 5 = absolutely certain).

Of note, due to the nature of the task, generalisation could only be assessed using the learned set of faces. Administering the same retrieval test twice, to obtain assessments of memory and generalisation at the start (baseline) and end of the retention interval, was opted against, as possible additional learning during immediate retrieval would make the measures at the two time points incomparable. Therefore, we opted for a single retrieval session at the end of the retention interval.

#### Spatial memory selection task

In view of the main tasks' difficulty, performance on a spatial memory selection task served as exclusion criterion. In this task, 20 colour photographs of everyday objects were each coupled to a chessboard location. Procedures were similar to the main tasks', except that the set of 20 object-location pairings was shown only once at encoding, and retention was tested after a 5-min interval, in which participants worked on sudoku. A minimum score of 13 correctly retrieved object locations was set as threshold to continue the experiment.

#### Procedure

Prior to the experiment, participants were randomly assigned to a sleep condition (*N* = 18) and a sleep deprivation condition (*N* = 24). Upon arrival at the lab, participants signed an informed consent statement, completed the PSQI, SQS, and SSS, and a demographic data questionnaire. At 8:30 p.m., they started with the spatial memory selection task. Subjects with a score below threshold were excluded from further participation. The remaining participants took a 15-min break and performed the main memory task at approximately 9:15 p.m. (duration of the task ± 45 min). Participants were instructed to memorise as many face-location associations as possible. No hints whatsoever were given about the presence of regularities in the material. After the completion of the task and a 15-min break, participants performed the PVT.

Participants in the sleep condition then prepared for bed and were given the opportunity to sleep from 11 p.m. (lights off) until 8 a.m. (no physiology recorded). At ~9.30 a.m. (after having breakfast, etc), they again completed the SSS, performed the PVT, and left the lab. The procedure for the sleep-deprived condition was identical to that of the sleep condition, except that subjects spent the night following encoding awake in the sleep lab, under the supervision of an experimenter. They were not allowed to watch content that contained faces to avoid interference. The next morning, subjects left the lab. They were instructed to take a 1.5-h nap between noon and 3 p.m. and follow their normal sleep routine during the ensuing two nights. Adherence to this sleep–wake recovery schedule was controlled through actiwatch (Cambridge Medical) registrations.

All participants returned to the lab 2 nights after their first visit, at 10 a.m. (memory retention interval: 60 h). They performed the retrieval part of the memory task, completed the SSS, and performed the PVT for the last time.

#### Data analysis

Spatial error in the retrieved location of each face was expressed as the Euclidian distance between the correct location and the retrieved location, calculated as follows (Pythagoras' formula):


$$\begin{array}{l} S\;=\sqrt{{\left(\frac{X\;correct\;-\;X\;recall}{80}\right)}^{2}+\;{\left(\frac{Y\;correct\;-\;Y\;recall}{80}\right)}^{2}} \end{array}$$

in which X correct and Y correct are the coordinates of the correct (learned) face location, X recall and Y recall are the coordinates of the retrieved location, and 80 is the length of one chessboard square in pixels. This placement error summed across all 64 faces constitutes the total spatial error per subject. In the final spatial error measure, this total error is divided by 64, to reflect the average spatial error per retrieved location.

**Figure 3:**
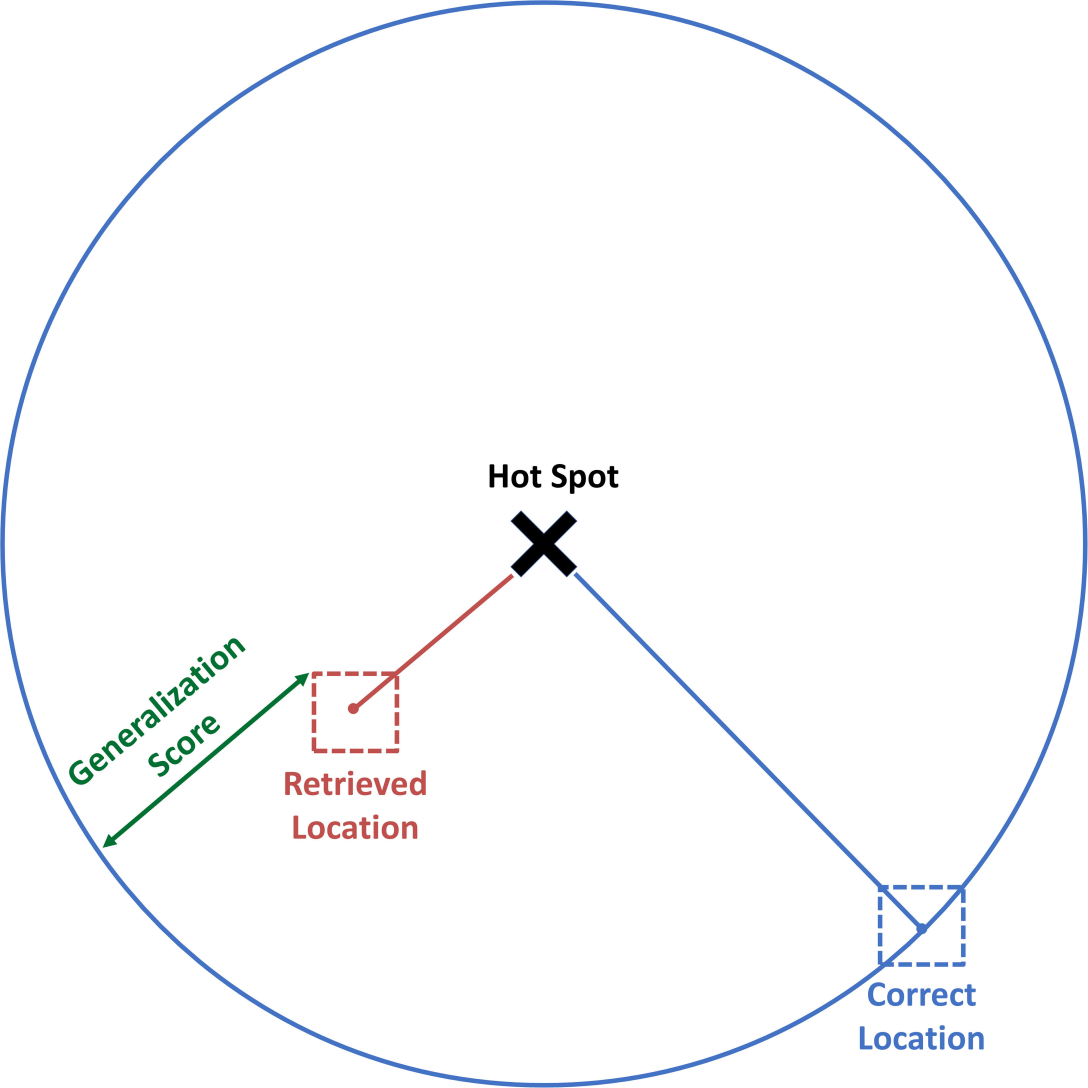
Operationalisation of generalisation. Generalisation for a single retrieved face location, R, was calculated by subtracting the distance of this location to the hotspot location, RH, from the distance of the correct face location to the hotspot, CH. Distances RH and CH were calculated with the Pythagorean equation. C, Correct location; H, Hotspot; R, Retrieved location.


Generalisation was operationalised as the deviation of spatial error in location retrieval towards the hotspots. First, generalisation for each retrieved location was calculated as the distance between the correct location and the hotspot location, minus the distance between the recalled location and the hotspot location (Figure 3), according to:

$$\begin{array}{l} D_{hotspot}\\ =\;\sqrt{{\left(\frac{X\;hotspot\;-\;X\;encoding}{80}\right)}^{2}+\;{\left(\frac{Y\;hotspot\;-\;Y\;encoding}{80}\right)}^{2}}\\ -\;\sqrt{{\left(\frac{X\;hotspot\;-\;X\;recall}{80}\right)}^{2}+\;{\left(\frac{Y\;hotspot\;-\;Y\;recall}{80}\right)}^{2}} \end{array}$$

For the final generalisation measure per participant, D_hotspot_ values were summed across all 64 items:


$$Gen_{tot}=\frac{\left(\sum Dhotspot_{1-64}\right)}{\left(64\right)}.$$

Of note, in case of directionally random error (i.e., no bias of error in any direction), Gen_tot_ would be slightly negative. Indeed, due to the non-random distribution of faces in each category over the field, random errors would tend to bring faces, on average, further from the hotspot. As the statistics to determine the value of Gen_tot_ reflecting directionally random error are complex, a simpler measure was used to determine whether generalisation occurred at all. Specifically, we assessed whether subjects had a positive generalisation score (i.e., any amount of deviation towards the hot spot location) for more items than expected based on chance. Chance level is given by:


$$\begin{array}{l} E\;\left(\chi \right)\;=\;\overset{64}{\underset{i=1}{\sum }}\frac{\chi_{i}}{64} \end{array}$$

in which E (χ) is the expected number of faces with a positive generalisation score out of all 64 faces, x_1_ is the probability of a positive generalisation score for face 1, x_2_ is the probability of a positive generalisation score for face 2, etc. E (χ) amounts to 13.23 faces.

**Figure 4:**
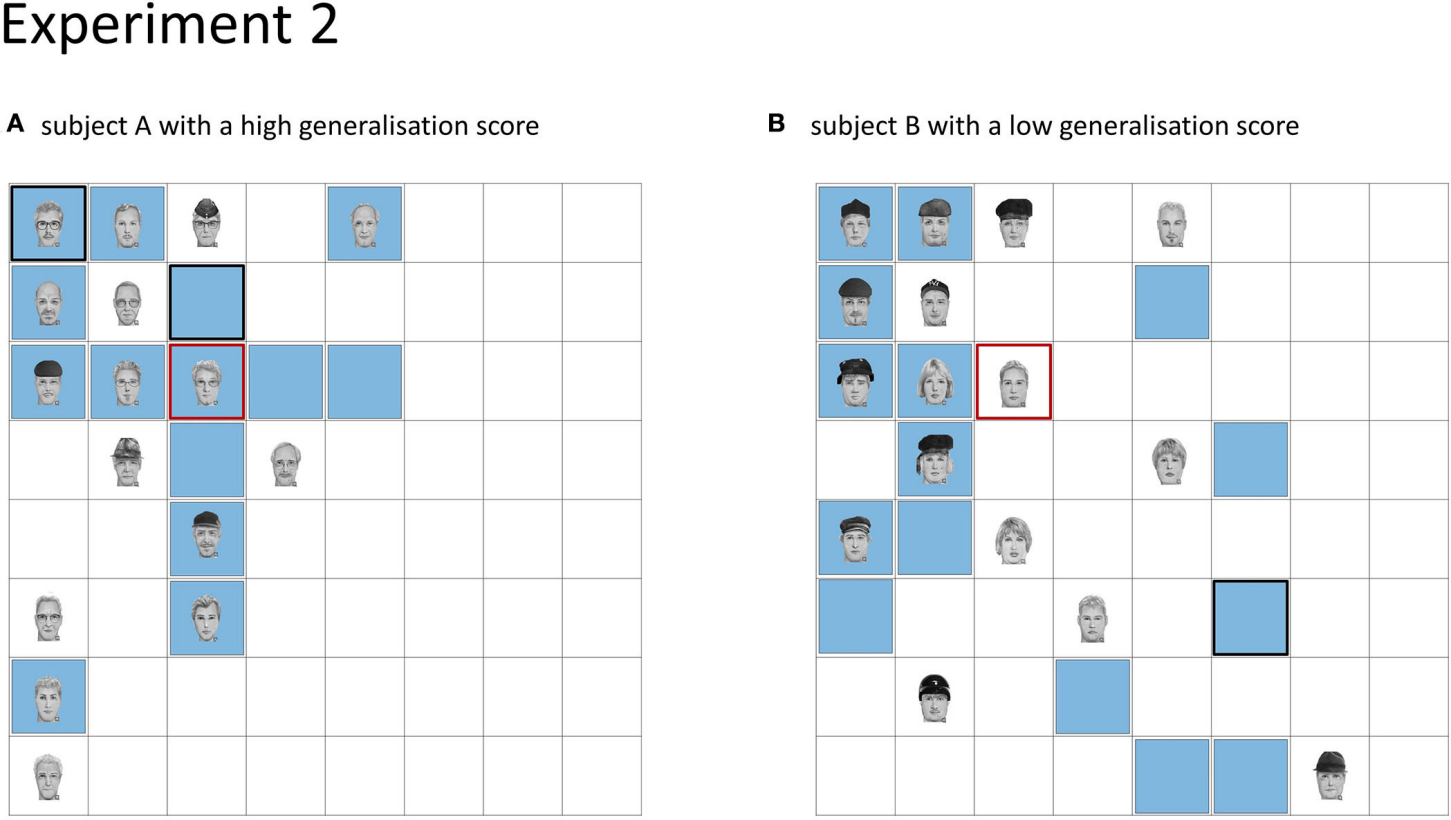
Depiction of generalisation score. **(A)** Subject with a high overall generalisation score (0.23). **(B)** Subject with a low overall generalisation score (−1.02). For each subject, one face category with the concomitant hotspot in the left upper quadrant of the board is shown. The red square shows the location of the hotspot, the faces show the learned face locations, and locations, where the subject placed a face during recall are indicated by blue squares. Locations, where the subject placed a face twice, are shown by a thicker black. For subject A with a positive generalisation score, recalled locations are closer to the hotspot.

Subsequently, the more sensitive measure Gen_tot_ was used to assess the difference in the amount of generalisation between the sleep and sleep deprivation groups, with values that are more positive indicating stronger overall generalisation (see Figure 4 for a visualisation of generalisation score).

All variables were tested for normality. For comparing the sleep group and the sleep-deprived group, an independent *t*-test was performed, on all the data that met the criteria for parametric testing. For the variables that did not meet the criteria of parametric testing, the Mann–Whitney *U* test was performed (exact). Moreover, in order to statistically rule out the presence of effects substantial enough to be regarded as valuable, we performed an equivalence test (Lakens, 2017), namely the two one-sided test (TOST). Using the TOSTER package in R (RStudio, 1.4.1717), we determined the upper and lower bounds as ±0.87, given α = 0.05 and 0.8 statistical power in a sample size equal to 46.

### Results

#### Experiment 1 (episodic regularity extraction across natural sleep and wake episodes)

##### Control measures

First of all, we controlled for circadian effects on performance in all components of the task, by comparing scores at immediate retrieval between groups. For the face-location and temporal order memory, this was done through 2-factor mixed ANOVA, with GROUP (sleep, wake) as a between-subjects variable and REGULARITY (rule, no-rule) as within-subjects variables; for generalisation performance, a *t*-test was used. No significant effects, nor trends, were found in any of the analyses (all *p*'s > 0.1).

##### Face-location memory

**Figure 5:**
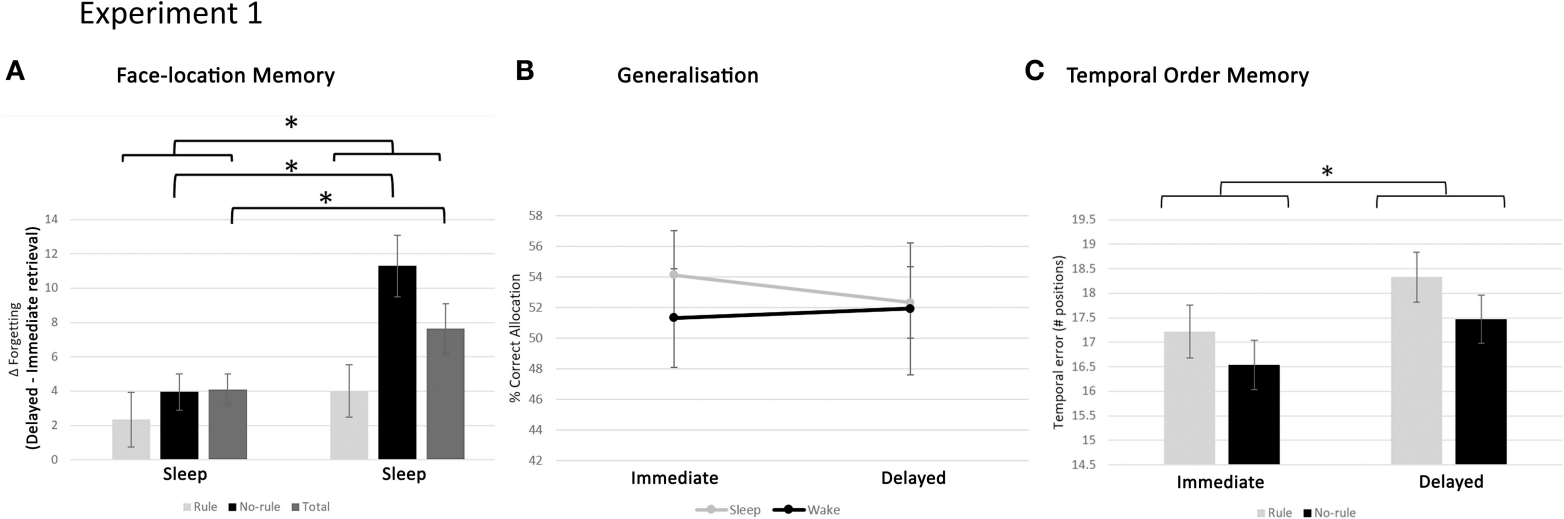
Results of experiment 1. **(A)** Forgetting rate for face locations, in the sleep and wake group, for the rule, no-rule, and total faces. Overall, retention is higher in the sleep group compared to the wake group and higher for rule items than no-rule items. Furthermore, no-rule items specifically are retained better in the sleep group than the wake group, suggesting sleep protects against episodic memory decay. **(B)** Correct allocation of new faces to screen locations, based on regularity knowledge, is indicated in percentage for the sleep and wake groups, directly after encoding (Immediate) and after 12 h (Delayed). Over the 12-h interval, performance remained stable for both the sleep and wake group, indicating no preferential benefit from sleep. **(C)** Performance on the temporal order task, expressed as temporal order error (number of positions away from the correct one, averaged over all faces), parsed for sleep–wake and rule/no-rule conditions. Chance level performance is 24. There is overall forgetting over the retention interval, with no notable difference between the sleep and wake condition. Temporal order memory is slightly better for no-rule faces than rule faces across the sleep and wake groups and retrieval sessions. Thus, the presence of regularities might negatively impact memory for episodic details. *p < 0.05. Error bars represent the standard error of the mean.

Average retrieval scores on the face-location task, for immediate and delayed retrieval, parsed out by group and regularity condition are given in Table 1. The data were analysed through ANOVA, with GROUP (sleep, wake) as a between-subjects variable and REGULARITY (rule, no-rule) and TIME (delayed retrieval, immediate retrieval) as within-subjects variables. As expected, retrieval accuracy was higher for rule items than no-rule items (main effect REGULARITY [*F*_(1, 35)_ = 45.30, *p* < 0.0005]) and decreased over time (main effect TIME [*F*_(1, 35)_ = 42.93, *p* < 0.0001]). Also, retention was, overall, better for rule items than no rule items (TIME × REGULARITY [*F*_(1, 35)_ = 10.87, *p* = 0.001; Figure 5A]) and higher in the sleep group than the wake group (TIME × GROUP [*F*_(1, 35)_ = 7.46, *p* = 0.021]). Importantly, there was also a significant interaction between GROUP, REGULARITY, and TIME [*F*_(1, 35)_ = 5.55, *p* = 0.025], reflecting better retention, for no-rule items specifically, in the sleep group compared to the wake group (Figure 5A). Thus, sleep does not benefit consolidation for regularities in the task, but rather protects purely episodic memory components from decay.

**Table 1:** Percentage of correctly recollected face-location associations at immediate and delayed testing in experiment 1 (mean percentage and SD).

		**12-sleep**	**12-wake**
Immediate recall	Rule	92.98 (8.75)	89.97 (10.83)
	No-rule	89.62 (9.84)	82.87 (10.83)
	Average	89.91 (9.11)	86.42 (11.69)
Delayed recall	Rule	90.64 (8.08)	85.96 (12.66)
	No-rule	85.67 (11.87)	71.60 (18.14)
	Average	85.82 (10.54)	78.78 (14.74)
*N*		19	18

##### Generalisation

In the generalisation task, subjects were presented with new faces, responding to the same regularities as the original learning set. These had to be allocated to screen location, using any previously acquired regularity knowledge. Overall performance on the task was well above chance level (16.7%), at both immediate retrieval (mean ± SD: 52.70 ± 12.99%) and delayed retrieval (mean ± SD: 52.14 ± 14.48%), showing that subjects were indeed able to extract regularities from the material and generalise the knowledge to new items.

Figure 5B shows generalisation performance for each group in the immediate and delayed test session. As can be seen in this figure, the level of performance was similar between groups and remained relatively stable over the 12-h interval. Indeed, ANOVA, with TIME (delayed retrieval, immediate retrieval) as a within-subjects variable and GROUP (sleep, wake) as a between-subjects variable showed no significant main (TIME [*F*_(1, 35)_ = 0.17, *p* = 0.68]; GROUP [*F*_(1, 35)_ = 0.06, *p* = 0.81]) or interaction (TIME × GROUP [*F*_(1, 35)_ = 0.07, *p* = 0.79]) effects. Thus, general knowledge remained relatively stable over a wake and sleep interval, with no preferential benefits from sleep.

##### Temporal order memory

The temporal order task investigated the faith of episodic elements that did not play a role in the formation of regularities. Performance on the temporal order task was expressed as a temporal error. That is, the deviation (in number of positions in a sequence of possible positions) from the correct order, averaged over all faces. The average temporal error for each group, in the immediate and delayed sessions, is given in Table 2. Statistical analysis was done through ANOVA, with GROUP (sleep, wake) as a between-subjects variable and REGULARITY (rule, no-rule) and TIME (delayed retrieval, immediate retrieval) as within-subjects variables. Results showed a significant effect of TIME [*F*_(1, 35)_ = 10.42, *p* = 0.003], in line with overall forgetting over the retention interval and a marginally significant effect of REGULARITY [*F*_(1, 35)_ = 3.79, *p* = 0.06], reflecting better temporal order memory for no-rule faces than rule faces, across groups and retrieval sessions (Figure 5C). Other effects were not statistically significant (all *p*'s > 0.1). Thus, forgetting arbitrary episodic details across the retention interval was not notably different between the sleep and wake conditions (see the Supplementary material for a violin plot visualisation of experiment one's results).

**Table 2:** Temporal order memory at immediate and delayed testing in experiment 1.

		**12-sleep**	**12-wake**
Immediate recall	Rule	17.70 (3.47)	16.71 (3.21)
	No-rule	16.33 (3.55)	16.77 (2.55)
	Average	17.02 (3.13)	16.74 (2.65)
Delayed recall	Rule	18.35 (3.22)	18.32 (3.09)
	No-rule	17.29 (3.15)	17.67 (2.82)
	Average	17.82 (2.88)	17.99 (2.46)
*N*		19	18

Values represent temporal error, i.e., the average nr of positions away from the correct one (mean and SD).

##### Trade-off between generalisation and memory for arbitrary detail

The relation between generalisation across task items and decay of arbitrary episodic details of individual items was further investigated through correlation analysis. To this purpose, a measure reflecting preferential decay of arbitrary details for no-rule items was calculated as: temporal order score for no-rule items minus score for rule items (collapsed over time). This variable showed a highly significant, positive correlation with performance on the generalisation task for the entire body of participants (*r* = 0.49, *p* = 0.005). After separating the sleep and wake group, an even stronger correlation was found within the wake group (*r* = 0.69, *p* = 0.006), but no significant correlation was found within the sleep group (*r* = 0.26, *p* = 0.31). This suggests a trade-off in the wake group, with larger decay of arbitrary details accompanying stronger generalisation.

#### Experiment 2 (effects of sleep deprivation on implicit regularity extraction)

##### Control measures

Vigilance and subjective alertness, assessed using the PVT and SSS, respectively, were not significantly different between the sleep and the sleep-deprived groups, either at the time of encoding or at recall (all *p*'s > 0.1). Thus, the findings in this study are not likely confounded by the effects of sleep-deprivation on alertness.

On day 2, i.e., the morning after a night of sleep or sleep-deprivation, the subjects filled out the SSS. As expected, the sleep-deprived group (Mdn = 4.0, Q1 = 3.5, Q3 = 5.0) felt significantly less alert than the sleep group (Mdn = 3.0, Q1 = 2.0, Q3 = 4.0; U = 96.5, *p* = 0.002) confirming successful sleep deprivation.

##### Face location memory

**Figure 6:**
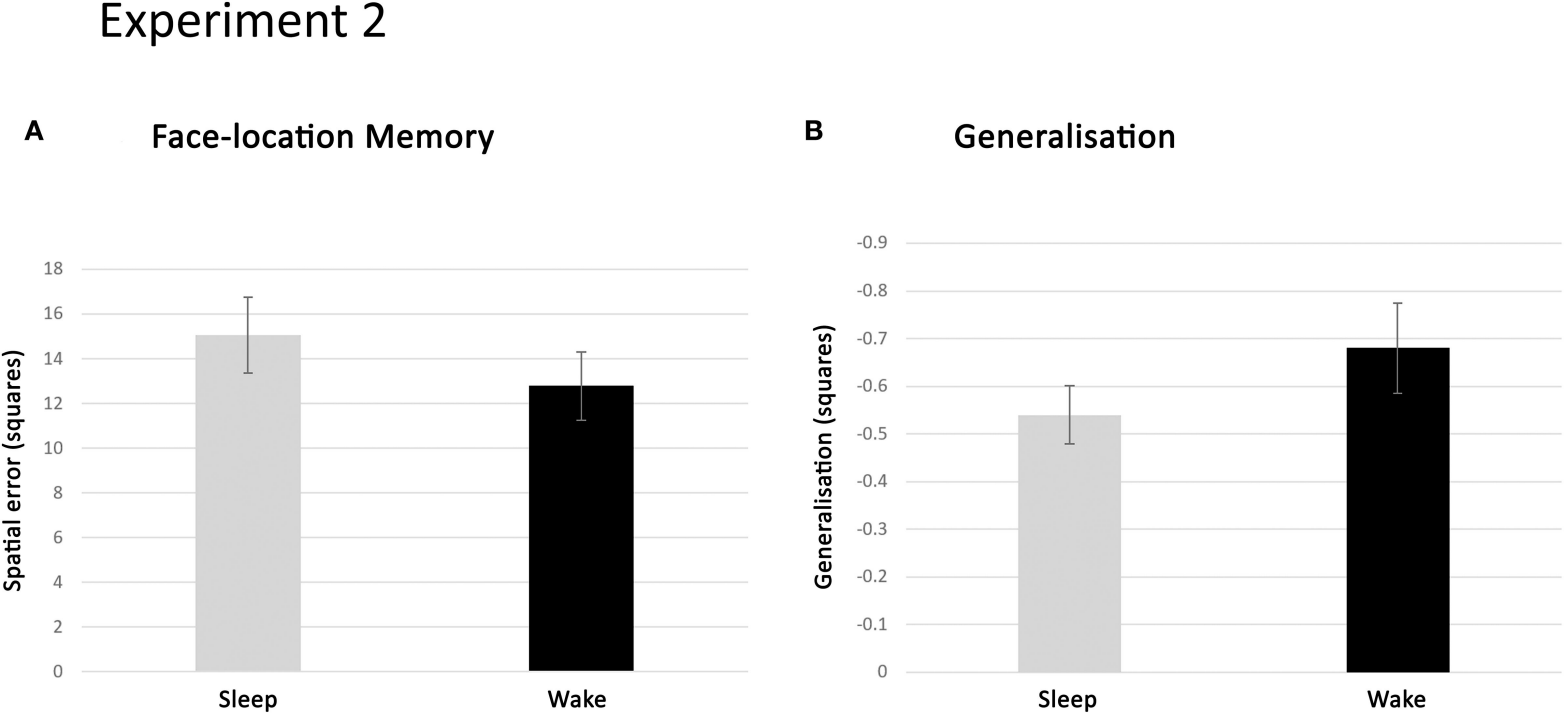
Results of experiment 2. **(A)** Spatial error in face location retrieval, in the sleep and sleep-deprived conditions, expressed as the number of positions away from the correct location, averaged across retrieved faces and subjects in each condition. Memory performance in the two conditions was similar (n.s.). **(B)** Generalisation in the sleep and sleep-deprived condition expressed as the spatial deviation (in number of positions) of face-location retrieval towards the hotspots, averaged across retrieved faces and subjects in each condition. Generalisation occurred to a similar extent in the two conditions (n.s.). Error bars represent the standard error of the mean.

Episodic memory accuracy in the chessboard task was expressed as the average spatial error across retrieved locations. That is, the distance between the correct and retrieved location averaged over all face-location items. As a second memory measure, the number of items placed exactly in the correct location was also considered. Participants in the sleep deprivation condition placed somewhat fewer faces correctly (mean 12.78, SD 7.90) than participants in the sleep condition (mean 15.05, SD 7.39) and showed a slightly larger spatial error (Figure 6A). However, neither of these differences reached statistical significance [*t*_(44)_ = 0.99, *p* = 0.33 and *t*_(44)_ = −1.48, *p* = 0.15, respectively]. A TOST equivalence test on these data render *p* = 0.031 for number of correctly placed faces and *p* = 0.078 for spatial error. This means a medium-sized effect of sleep vs. wake can be ruled out for the number of faces, but not for the spatial error measure.

##### Generalisation

Generalisation was operationalised as the average deviation of spatial error towards the hotspot. That is, the average distance between the correct location of a given face and the hotspot location minus the distance between retrieved location and hotspot location (Figure 3).

Based on these scores, we first analysed whether generalisation did, in fact, occur to any significant extent. To this purpose, we assessed whether subjects had a positive generalisation score (i.e., deviated towards the hot spot location) for more items than expected based on chance. Chance level amounts to a positive generalisation score for 20.7% of faces. In fact, participants, who collapsed over the sleep and sleep deprivation conditions, had a positive generalisation score for, on average, 26.4% of faces, which is significantly higher than the chance level [*t*_(44)_ = 7.29, *p* < 0.0005]. This indicates that participants were indeed able to extract the associative regularities embedded in the face-location task and that this general information-biased retrieval responses.

Having established this, we went on to investigate whether sleep in the night after encoding influenced generalisation. As a first indication, participants from both conditions generalised on a similar percentage of faces [sleep: 26.0%, sleep deprivation: 26.9%; *t*_(44)_ = −0.53, *p* = 0.60]. The amount of generalisation was, on average, somewhat higher for participants in the sleep condition (mean −0.54, SD 0.27) than those in the sleep deprivation condition (mean −0.68, SD 0.49; Figure 6B). However, this difference was not statistically significant [*t*_(44)_ = 1.14, *p* = 0.26]. TOST test of equivalence on this data gives a *p* = 0.042, suggesting we can rule out a medium-sized effect of sleep on generalisation (for a visualisation of dispersion in the data of experiment 2, see the Supplementary material).

To control the possible impact of response confidence on generalisation, a second analysis was performed using a linear mixed model, with certainty (5 levels, from very uncertain to very certain) and group (sleep, sleep-deprived) and their interaction (certainty^*^group) as independent variables, participants as a random effect and generalisation score as the dependent variable. The analysis outcome shows a strong positive relation between confidence and generalisation (*F* = 17.041, df = 4, 2766.8, *p* < 0.001); that is, generalisation occurs more strongly for items that were placed more confidently. Confidence does, however, not interact with Group (*F* = 0.942, df = 4, 2766.8, *p* = 0.438). Accordingly, the difference in generalisation between groups was again not significant (*F* = 1.95, df = 1, 59.3, *p* = 0.168). A full motivation and description of this analysis are given in the Supplementary material.

Finally, as the distance over which generalisation bias can potentially occur is different for each face on the chessboard task, generalisation was also calculated as a proportion of the distance of each face from the respective hotspot location (i.e., the generalisation measure was divided by the distance between the faces correct location and the hotpost location). Again, no significant difference between the sleep and sleep-deprived group was found with this proportional generalisation score [*t*_(44)_ = −1.47, *p* = 0.148]. A more extensive account of this analysis is given in the Supplementary material.

The combined results suggest that the first night of sleep after exposure to an episodic memory task holding hidden regularities does not significantly affect the extent to which the regularities are extracted and implicitly influence task performance.

## Discussion

### Summary of results

The presented studies investigated the abstraction of associative regularities across episodic exemplars and the role of sleep in this process. In the first experiment, we assessed the retention of memories for associative regularities and arbitrary associations over 12-h post-learning intervals containing sleep or wakefulness. Retention of arbitrary associations was facilitated by sleep specifically. In contrast, associative regularities and generalisation performance were strongly retained over sleep and wake time alike. A second experiment used a paradigm involving sleep deprivation on the first night after learning to address the causal contribution of sleep to the consolidation of episodic regularities, focusing on implicit effects. Again, generalisation occurred, but no significant effect of the sleep–wake manipulation on generalisation was observed. Finally, the results of the first experiment show that regularity extraction was negatively associated with the storage of unique, regularity-irrelevant episodic memory components. Hereafter, we will discuss these findings in more detail.

### The tasks

To ensure that regularity extraction would depend on the storage of episodic memories, we employed tasks that involved the encoding of multiple associations, in particular, between faces and their spatial location. Such tasks have been shown to call strongly upon hippocampal functioning (Giovanello et al., 2003; Zeineh et al., 2003; Staresina and Davachi, 2009; Takashima et al., 2009; Westerberg et al., 2012). The regularity structure embedded in the learning material of both tasks was highly complex to ensure slow and gradual development of regularity knowledge, across many exemplars, and to allow for further offline development after the learning phase. In view of this complexity, the contingencies making up the regularity structure were only very partially grasped by subjects, at any time, during either experiment. This is reflected by the generalisation scores that remained around 50% in experiment 1, while in experiment 2 generalisation (error bias conforming to the regularity structure) occurred for only 26–27% of faces.

The hexagonal board task, used in experiment one, is highly similar to the task used in our previous study that assessed generalisation across episodes using a nap design (Sweegers and Talamini, 2014). In that study, we used a regularities questionnaire to assess how subjects' explicit knowledge of the regularity structure developed across the experiment. The results showed that, in the course of the learning rounds, subjects gradually achieved some explicit understanding of which might be the rule locations and of some of the facial features that might be associated with each location (being able to indicate on average 2.3 of the rule locations and 1.1 of the facial features associated to each location). This explicit insight developed further across the 4-h retention interval along with the increase in generalisation performance. These previous studies support the notion that at least part of the regularity extraction that occurs during the hexagonal board task is explicitly accessible. The task used in experiment 2 was developed *de novo* for the purpose of assessing implicit components of cross-episodic regularity extraction. The task design is based on the idea that any available regularity knowledge would unconsciously bias recall responses towards the regularity structure, especially in case of uncertainty. Specifically, imperfect episodic memory would be associated with episodic memory errors, which would be biased towards the spatial organisation of facial features.

### The role of sleep in generalisation

The current experiments were designed to complement and extend a previously reported study, in which cross-episodic generalisation was assessed over a 4-h interval containing a wake or a nap (Sweegers and Talamini, 2014). Combining this previous study with our current two studies, cross-episodic generalisation developed similarly over post-encoding sleep and wake intervals. All the experiments had samples per design cell between 16 and 26 subjects, and the tasks assessing cross-episodic generalisation were sensitive enough to robustly detect generalisation over time, in all three experiments. The design of the three studies was complementary, such that together they address consolidation intervals of varying duration (4, 12, and 60 h) and use different sleep–wake manipulations (nap, full night of sleep, and sleep deprivation). Potential confounds of any individual study, such as time of day effects on retrieval or dilution of differential sleep–wake effects in recovery nights, are not present in the other two. This does not fully exclude alternative explanations of the findings regarding sleep but makes them highly unlikely.

The tasks adopted in the three studies also differed. Most importantly, the hexagonal board task and the chessboard task were developed to assess explicit and implicit regularity knowledge, respectively. In addition, the two tasks differ with regards to the acquisition paradigm (with memory testing during the acquisition in the hexagonal board task; without in the chessboard task) and with regards to item overlap (multiple faces share one location in the hexagonal board task, all face-location items are unique in the chessboard task). Despite these differences, the results are consistent across the two experiments, favouring the notion that sleep and wake time are similarly important for regularity extraction across episodes. Of course, we cannot conclude based on null-findings that cross-episodic generalisation is entirely indifferent to sleep–wake states. However, our findings suggest that, if differences exist, they will be small or unreliable.

Interestingly, a recent study by another lab also assessed the role of sleep on generalisation in a task with episodic memory features (Chatburn et al., 2021). As in our studies, generalisation did not significantly depend on post-learning sleep–wake conditions. While the above studies all focus on the extraction of cross-episodic regularities over multiple exemplars, another study addressed a parallel mechanism contributing to memory abstraction, namely the loss of contextual information from episodic memories. In this study with 79 participants, decontextualisation of memories was assessed over 12- and 24-h intervals beginning with either sleep or wakefulness. The results of the study suggest that memories decontextualise over time, but independent of its sleep and wake content (Cox et al., 2014b).

Of note, our findings by no means imply that sleep has no role in generalisation across episodes. In fact, our previous observation (Sweegers and Talamini, 2014) of an increase in generalisation performance and explicit regularity knowledge in the 4 h post-encoding suggests that some active generalisation process continues after exposure to episodic exemplars. In the pertaining study, this generalisation process correlated with time spent in SWS in the sleep group. This is perhaps not surprising, given a large body of evidence linking memory reactivation and reprocessing to this sleep stage (Diekelmann and Born, 2010) and its oscillatory hallmarks: slow waves (Mölle et al., 2004; Marshall et al., 2006) and spindles (Gais et al., 2002; Cox et al., 2012, 2014a). Having said this, our findings show that similar generalisation occurs over time spent in wake. This might be related to observations in rodents showing that hippocampocortical replay occurs not only during sleep but also during awake rest and consummatory behaviours (Foster and Wilson, 2006; Davidson et al., 2009; Karlsson and Frank, 2009). Moreover, a study in humans (Sawangjit et al., 2022) points to wake-associated consolidation that supports context-independent memory for events/objects. These findings, while not negating the role of sleep in generalisation, do point to a substantial role of wake time in such processes.

### The temporal course of cross-episodic regularity extraction

While many studies have investigated the processing of regularities in presented materials, few studies have capitalised on the processing of regularities that can only be extracted across hippocampus-dependent memories (Sweegers and Talamini, 2014; Sweegers et al., 2014). Taken together, these studies, mostly from our lab, give some information about the temporal course of cross-episodic regularity extraction. As indicated previously, we performed three complementary studies featuring consolidation of face-location regularities over intervals of 4 (Sweegers and Talamini, 2014), 12, and 60 h (current experiments 1 and 2), and 1 month (Sweegers and Talamini, 2014). At all time points, retention of regularities was assessed through generalisation performance, providing a pure measure of regularity knowledge. Considering our previous experiment (Sweegers and Talamini, 2014; Sweegers et al., 2014) and current experiment 1, it appears that generalised knowledge builds up to a considerable extent during exposure to episodic exemplars. Indeed, a difference in performance between “rule” and “no-rule” items builds up gradually with each training round. At the end of training, subjects are able to successfully generalise extracted regularity knowledge to new items and, as observed in Sweegers and Talamini (2014), generalisation performance further increases between the end of the encoding session and 4 h after exposure. Thereafter, regularity knowledge appears to remain remains highly stable. Indeed, no change in generalisation is seen over the 12-h retention interval (perhaps suggesting slight forgetting may occur after the first 4 h post-encoding) and, even at 1-month post-encoding, no significant decay of regularity knowledge was observed. This is strikingly different from the retention of unique episodic components which decay at a rapid rate. Indeed, in the same study (Sweegers and Talamini, 2014), retrieval performance for unique face-location items decreased by about 60% in the same period. The differential retention curves for regularity knowledge and unique episodic memory components are in line with the broad literature on long-term memory, showing fast decay of arbitrary, hippocampus-dependent memory components and relative stability of semantic memory and other forms of general knowledge (McClelland et al., 1995; Martin and Chao, 2001; Frankland et al., 2004; Meeter and Murre, 2005; Moscovitch et al., 2005; Binder et al., 2009; Winocur et al., 2010; Battaglia and Pennartz, 2011; Talamini and Gorree, 2012; Rasch and Born, 2013).

### Effects of generalisation on storage of arbitrary episodic details

As an ulterior research question, we asked whether cross-episodic memory extraction would influence the storage of arbitrary episodic memory components. This was addressed through memory for items' temporal order, which is known to be dependent on the hippocampus for encoding (Mayes et al., 2001; Warburton and Brown, 2015; Long and Kahana, 2019). Temporal order tended to be stored less well for items reflecting regularities compared to unique ones, during both immediate and delayed retrieval (without a significant change across the consolidation interval). Furthermore, generalisation performance was associated with poor storage for arbitrary detail in a correlation analysis. A similar observation was made in our previous study (Sweegers and Talamini, 2014), where we found reduced storage of facial details for exemplars responding to regularities. These findings are in line with reduced encoding and/or very early consolidation of arbitrary elements. A tentative interpretation of these results holds that attention and subsequent neural processing are biased towards the overlapping aspects of episodes. Given capacity limits, especially in working memory (Turner and Engle, 1989; Luck and Vogel, 1997), this would likely lead to suboptimal processing of the remaining aspects of the material. Supporting this notion, we have previously shown that pre-existing regularity knowledge (i.e., established prior to task onset) hinders the in-depth processing of knowledge-congruent novel input, thereby impairing the formation of perceptually detailed and contextually rich memory traces (Sweegers et al., 2015).

### Effects of sleep on retention of arbitrary episodic details

The trade-off between generalisation and memory for arbitrary details appeared to be driven particularly by the wake group. This may relate to the fact that encoded arbitrary item features were protected and retained by sleep, while in the wake group, these details suffered stronger forgetting. Indeed, while no major effect of sleep–wake state was found on the consolidation of regularities, our data suggest that sleep did specifically benefit the retention of no-rule face-location associations. This observation is in line with a large number of studies reporting higher episodic and declarative memory retention over sleep compared to wake intervals (Ellenbogen et al., 2006, 2009; Talamini et al., 2008). Within the boundaries of this study, it is not clear whether this is the consequence of some sleep-related consolidation process or a passive consequence of low interference (Yonelinas et al., 2019). Indeed, a recent review claims such doubt applies to the combined body of human studies assessing the effects of sleep on declarative memory retrieval (Cordi and Rasch, 2021). Nevertheless, theoretical studies have shown how sleep-related replay of neuronal memory representations, which has convincingly been demonstrated, could contribute to continual learning by combining consolidation of new memory traces with recoding and reconsolidation of old memory traces to minimise interference and prevent catastrophic forgetting (Káli and Dayan, 2004; González et al., 2020). In modelling studies, such mechanisms tend to improve retrieval of both new and old memories (González et al., 2020). From a minimal utility perspective, the increased retention of arbitrary episodic memories during sleep would benefit cross-episodic generalisation indirectly by keeping these episodes available for a longer time for cross-episodic regularity extraction.

### Related findings

Inspired by notions of memory consolidation and transformation during sleep, a large number of studies have addressed the role of sleep in the extraction of regularities from wake experiences. These studies, using a wide variety of tasks that likely engage many different memory systems and brain areas, have produced widely differing results. As observed in a recent review (Lerner and Gluck, 2019), findings seem consistent among experiments using a particular (type of) task, but different between studies using different tasks. Given the putatively different neural underpinnings of generalisation-type processes in different tasks, this should perhaps not be surprising. After all, regularity extraction is a basic feature of neural processing, occurring at all levels of the nervous system, in pathways that differ extensively with regards to neurophysiology and computations performed. The cognitive phenomenology associated with these different types of computations, including the influence of sleep and wake states, may therefore also differ.

The literature on regularity extraction is further complicated, because some of the tasks that have been used, likely depend on processes besides regularity extraction. For instance, many studies employ tasks that, besides regularities in the material, feature an “insight” component. In such studies, subjects are exposed to material embedding hidden regularities that, if discovered, suddenly and strongly improve performance (Gómez et al., 2006; Ellenbogen et al., 2007; Tamminen et al., 2012; Durrant et al., 2013). Such tasks are thought to require creative problem-solving; that is, thinking beyond obvious solutions by flexibly recombining memory and knowledge elements in innovative ways (Kounios and Beeman, 2014; Gilhooly, 2016). Sleep may well favour this kind of processing, given several characteristics that facilitate cross-activation among weakly associated representations in neural networks (Braun, 1997; Stickgold et al., 1999; Sil'kis, 2009; Wierzynski et al., 2009; Lewis et al., 2018). Furthermore, several studies have reported the benefits of sleep for creative problem-solving, even in the absence of regularity extraction (Walker et al., 2002; Wagner et al., 2004; Cai et al., 2009; Gupta et al., 2010; Verleger et al., 2013; Beijamini et al., 2014; Perdomo et al., 2018), but see also Schönauer et al. (2018). However, whether such processes are to any extent related to those serving gradual cross-episodic regularity extraction and hippocampo-cortical recoding, is currently an open question.

A recent review on this topic (Lerner and Gluck, 2019) concludes that, from research thus far, no clear consensus emerges regarding the aspects or conditions of regularity processing that yield a preferential effect of sleep. However, sleep does seem to facilitate extraction of hidden regularities when it depends on former knowledge (particularly semantic knowledge) that is likely not hippocampally dependent. This observation is in line with our findings showing no preferential effect of sleep in a generalisation task that is not dependent on any prior (semantic) knowledge.

Looking forward, we argue that advance in the field of sleep and regularity extraction would benefit from studies narrowing down hypotheses about the differential role of sleep–wake states to specific, well-delineated cognitive functions or to specific neural computations attributed to specific brain areas or neural networks. Experimental tasks and approaches should be designed to be as specific as possible to these functions or computations.

### Limitations and considerations

Considering the limitations of the current investigation, we would first like to reiterate that the tasks used in these experiments were designed to investigate a specific form of generalisation, namely the extraction of associative regularities across episodic exemplars. As argued in the previous section, we feel the findings should not be taken to apply to forms of generalisation or rule learning that may rely on other memory systems.

With regards to the chessboard task, it might be mentioned that, while the aim of constructing a task that can assess implicit aspects of episodic generalisation seems to have succeeded, there are certain intricacies to the task and the main generalisation measure. For instance, the maximum possible generalisation score differs for each item, depending on its correct location on the board. Analyses in fact show that generalisation scores tend to be higher for the items that are further from the hotspot (see Supplementary material). Given this circumstance, analyses considering subsamples of task items, or parts of the task, should be avoided. Further considerations regarding the chessboard task, including alternative ways to calculate generalisation scores (which do not notably change the results), are given in the Supplementary material.

It might, finally, be noted that neither of the two experiments involved polysomnography. Such recordings would have allowed analyses to explore the neural correlates of sleep-related generalisation (as in the Sweegers and Talamini, 2014 study), for instance in terms of spindle activity. As it is, such investigations will have to be left for future studies.

### Conclusion

In conclusion, our current and prior studies (Cox et al., 2014b; Sweegers and Talamini, 2014) suggest that the formation of higher-order general knowledge across episodic memories occurs to a similar extent over post-encoding sleep and wake intervals. Minor differences between the sleep–wake states cannot be excluded and might be revealed in studies with larger sample sizes. Nevertheless, these findings shed doubt on a long-standing and widely accepted hypothesis that states a preferential role of sleep in the formation of general knowledge across event memories.

Our results, furthermore, corroborate a large body of previous findings showing preferential retention of arbitrary episodic components during sleep, but warn that such findings should not be taken to imply a role of sleep in the transformation of memories. Finally, we have shown previously that cross-episodic knowledge builds up, to a large extent, during exposure to related episodes and in the hours thereafter, and remains strikingly stable thereafter, while memory for unique higher-order associations rapidly decays (Sweegers and Talamini, 2014). All in all, our combined studies provide comprehensive data on processes underlying episodic memory abstraction with important implications for theoretic models of sleep and memory.

## Data availability statement

The raw data supporting the conclusions of this article will be made available by the authors, without undue reservation.

## Ethics statement

The studies involving human participants were reviewed and approved by Ethics Committee of the University of Amsterdam. The patients/participants provided their written informed consent to participate in this study.

## Author contributions

LT designed and supervised both experiments and wrote the manuscript. RB and MB conducted and analysed experiment 1. DM and SS conducted and analysed experiment 2. MS helped with the literature search, drafting of the manuscript, (re)creating figures and additional analyses asked by the reviewers. MD assisted with data collection. All authors contributed to the article and approved the submitted version.
