# Supplementary material for: No evidence for a preferential role of sleep in episodic memory abstraction
Source: Front Neurosci. 2022 Dec 9;16:871188. doi: 10.3389/fnins.2022.871188 (PMC9780604; doi:10.3389/fnins.2022.871188)
Supplement: Supplementary file 1 [file Data_Sheet_1.PDF]

Supplementary materials

1 Supplementary considerations and analyses regarding the chessboard task

1.1 Putative interactions of episodic and regularity representations in the chessboard task

The way the chessboard task is envisaged (see the manuscript introduction and methods section), the individual face-location items require the hippocampus to form enduring representations. One might speculate, based on previous experimental literature and modeling accounts (see introduction of the main text), that representations of the regularities across these episodes are extracted in some process requiring hippocampo-prefrontal interaction (as in Sweegers et al., Neuroimage 87, 2014) and that existing (medio)prefrontal regularity representations then exert influence during the recall process. In such an account, the spatial bias toward the regularity pattern would itself not be represented, but would emerge due to influence of the regularity representations on recall. While in the broader literature it is widely acknowledged that episodic and semantic memory interact during encoding, consolidation and retrieval (Fang et al., 2018; Greve et al., 2007; Menon et al., 2002; Snowden et al., 1996; Takashima et al., 2017; Weidemann et al., 2019). The precise nature of this interaction remains poorly understood.

1.2 Relation between episodic memory and generalisation

Considering the relation between episodic memory and generalisation in the chessboard task (experiment 2), it should first of all be considered that discovering the regularity structure in the material is dependent on the formation of episodic memories for the individual face-location items. After all, the regularities have to be extracted across 64 individual face-location pairings presented sequentially and randomly across face-categories (see the main text, section ‘The Task’ in the discussion, for a more extensive account).

To investigate the relation between episodic memory and generalisation bias quantitatively, we correlated the episodic memory measure (spatial error) and the generalisation measure (spatial bias towards the hotspot location). This yields a moderate to strong invers correlation, (Pearson’s $r=-0.672$, $p<0.001$; see the figure below). Here, we have to consider that the episodic memory measure is a spatial error measure. Thus, better episodic memory performance is indeed related to higher generalisation scores, as expected based on theoretical considerations.

Experiment 2

Generalisation and temporal error

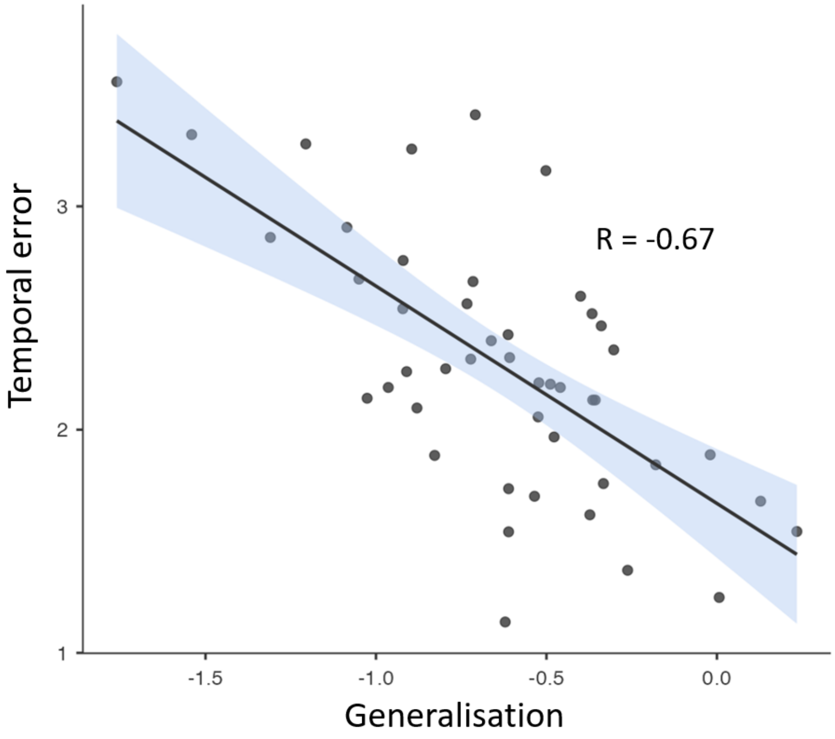

1.3 Relation between episodic memory response confidence and generalisation

In the following we will consider the relation between episodic and regularity memories in the chessboard task at the level of individual items. Inherent to the chessboard task, some level of uncertainty in episodic memory is necessary to assess the influence of regularity representations on performance. After all, generalisation bias can only be assessed in terms of the error in location retrieval for imperfectly placed faces. Given this characteristic of the task, we recalculated the overall generalisation score per participant, excluding faces for which generalisation is, by definition, not possible, i.e. correctly placed faces and the faces belonging on the hotspot locations. This modified generalisation measure might be slightly more sensitive and is calculated as:

$$Gen_{tot} = \frac{(\sum Gen_{1-i})}{(64 - \#correctly\ placed\ faces\ - \#incorrectly\ placed\ hotspots)}.$$

In which $Gen_{1\text{-}i}$ only considers incorrectly placed faces that are not hotspot faces.

The results of the analysis are very similar to the original one. The amount of generalisation was, on average, somewhat higher for participants in the sleep condition (mean -0.55, SD 0.34) than those in

the sleep deprivation condition (mean -0.69, SD 0.44), but the difference was not statistically significant (t (44) = 1.14, p = 0.26).

Beyond this circumstance, it is difficult to hypothesize how episodic and regularity representations might interact, as there are very few studies on this subject (see the main text for a discussion thereof). Possibly, recall of face locations with a very strong episodic representation may be less susceptible to influence of regularity representations. On the other hand, items with very weak episodic representations could lead to random face placement during recall, leading to large spatial errors, which - in turn - would introduce a large random component in the generalisation measure (which indicates bias from the original face location towards the hotspot).

In view of the above, response certainty for face location recall was assessed during the chessboard task, as a proxy for the strength of the episodic memory representation of a particular item. This allowed us to assess how response certainty relates to generalisation and the role of sleep in generalisation. We analysed these relations using a linear mixed model (LMM), with Participants as a random effect and Certainty (5 levels, from very unsure to very certain) and Group (Sleep, Sleep-deprived) and their interaction (Certainty*Group) as independent variables. The dependent variable was the generalisation score. The analysis outcome shows a strong positive relation between confidence and generalisation (F=17.041, df 4, 2766.8, p< .001); that is, generalisation occurs more strongly for items that were placed more confidently. Confidence does, however, not interact with Group (F=0.942, df 4, 2766.8, p= 0.438). Accordingly, the difference in generalisation between groups was again not significant (F=1.95, df 1, 59.3, p= 0.168). The complete results of the LMM are given below.

The positive relation between confidence and generalization may reflect that items placed with (very) low uncertain are unreliable. Therefore, even though the LMM did not indicate an interaction between Confidence and Group, we compared generalisation in the sleep and sleep deprived groups again, after excluding items with low certainty scores. Of note, after eliminating these datapoints the data was no longer normally distributed, thus comparisons were done using the Mann-Whitney U test. Excluding data points with the lowest certainty score did not indicate a significant difference between groups (U=191.0, p=0.53), nor did an analysis excluding the two lowest certainty scores (U=204.0, p=0.77). As such, taking response confidence into account did importantly alter the results of our analyses, or the conclusions regarding our main hypothesis on the role sleep in generalisation across episodic memories.

Mixed Model

Model Info

Info	
Estimate	Linear mixed model fit by REML
Call	Generalization ~ 1 + Group + Confidence + Group:Confidence+(1 | PP)
AIC	12056.1281
BIC	12150.9231
LogLikel.	-6027.5364
R-squared Marginal	0.0287
R-squared Conditional	0.0425
Converged	yes
Optimizer	bobyqa

Model Results

Fixed Effect Omnibus tests

	F	Num df	Den df	p
Group	1.953	1	59.3	0.168
Confidence	17.041	4	2766.8	< .001
Group ✻ Confidence	0.942	4	2766.8	0.438

Note. Satterthwaite method for degrees of freedom

Fixed Effects Parameter Estimates

Names	Effect	Estimate	SE	95% Confidence Interval		df	t	p
				Lower	Upper			
(Intercept)	(Intercept)	-0.4895	0.0526	-0.5926	-0.3864	59.3	-9.306	< .001
Group1	Sleep-deprived - Sleep	-0.1470	0.1052	-0.3532	0.0592	59.3	-1.397	0.168
Confidence1	2 - 1	0.3901	0.0956	0.2028	0.5775	2720.3	4.082	< .001
Confidence2	3 - 1	0.3710	0.1023	0.1706	0.5714	2381.5	3.628	< .001
Confidence3	4 - 1	0.7936	0.1096	0.5788	1.0084	2831.2	7.240	< .001
Confidence4	5 - 1	0.7708	0.1447	0.4872	1.0544	2792.5	5.328	< .001
Group1 ✻ Confidence1	Sleep-deprived - Sleep ✻ 2 - 1	0.1632	0.1912	-0.2115	0.5378	2720.3	0.854	0.393
Group1 ✻ Confidence2	Sleep-deprived - Sleep ✻ 3 - 1	-0.0934	0.2045	-0.4943	0.3074	2381.5	-0.457	0.648
Group1 ✻ Confidence3	Sleep-deprived - Sleep ✻ 4 - 1	0.3312	0.2192	-0.0985	0.7608	2831.2	1.511	0.131
Group1 ✻ Confidence4	Sleep-deprived - Sleep ✻ 5 - 1	0.1154	0.2894	-0.4517	0.6825	2792.5	0.399	0.690

Random Components

Groups	Name	SD	Variance	ICC
PP	(Intercept)	0.224	0.0501	0.0143
Residual		1.861	3.4636	

Note. Number of Obs: 2944 , groups: PP 46

1.4 Proportional generalisation

Given that faces' correct locations are differently spaced with respect to the hotspot location, the distance over which bias toward the hotspot can occur, also differs across faces. Thus, the maximal generalisation score is larger as the correct location is further away from the hotspot. Given this circumstance, generalisation could also be expressed as a proportion of the distance from correct location to the hotspot. Such a measure would be particularly interesting if generalisation indeed tends to be larger for more distant correct locations. To evaluate whether the distance between correct location and hotspot affects the generalisation score, we first did a General Linear Model analysis, with the correct location's distance from the hotspot as a covariate and the generalisation score as a dependent variable. The results indicated a significant positive correlation between these measures (R= 0.486, P<. 001), suggesting that generalization tends to be stronger for the items that are further from the hotspot.

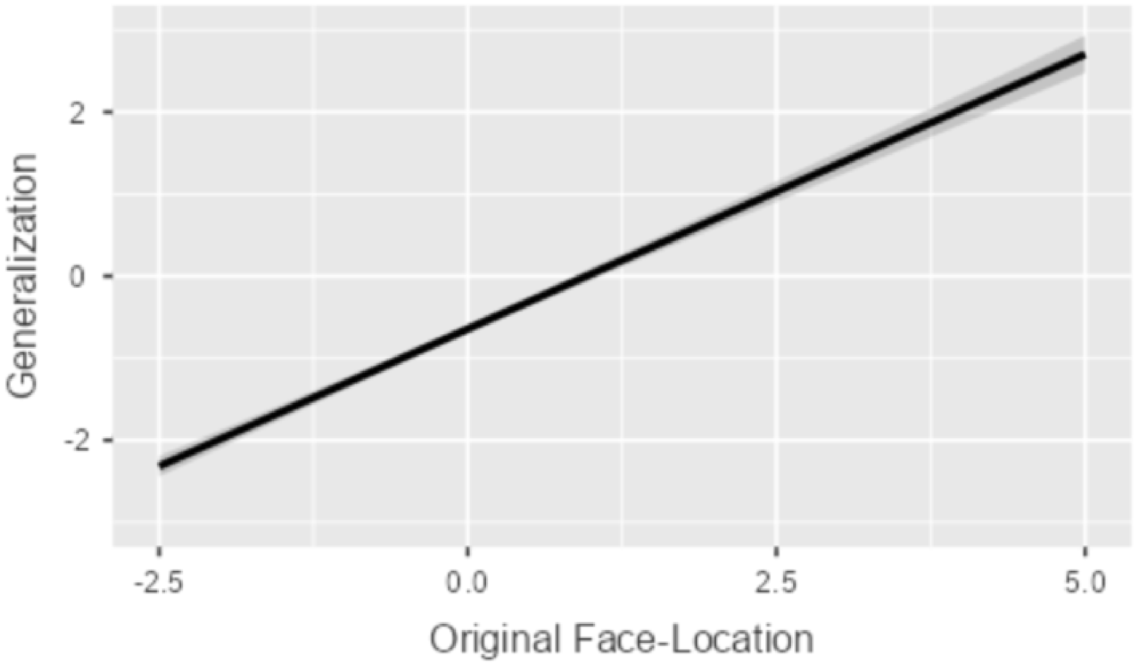

Given this correlation, we then calculated a new generalisation metric (proportional generalisation) by normalising the current generalisation score for the distance of the correct face location to the hotspot. To do so, we divided the current generalization score by the distance between the original location and the hotspot. Then, we analysed the data using a Linear Mixed Model with subjects as a random component, Groups (sleep, sleep-deprived) as the independent variable, and proportional generalisation as the dependent variable. However, still, no significant difference between the sleep and sleep-deprived group was found with this proportional generalisation score (t(44)=-1.47, p=0.148).

2 Supplementary figures for the results of experiments 1 and 2

Experiment 1

(A) Face-location Memory

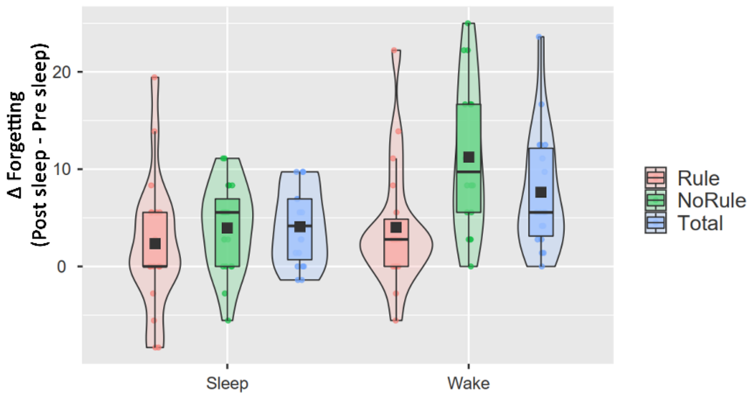

(B) Generalisation

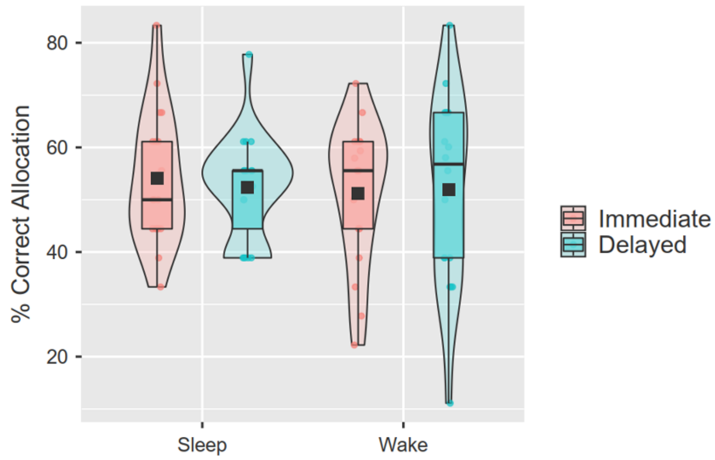

(C) Temporal Order Memory

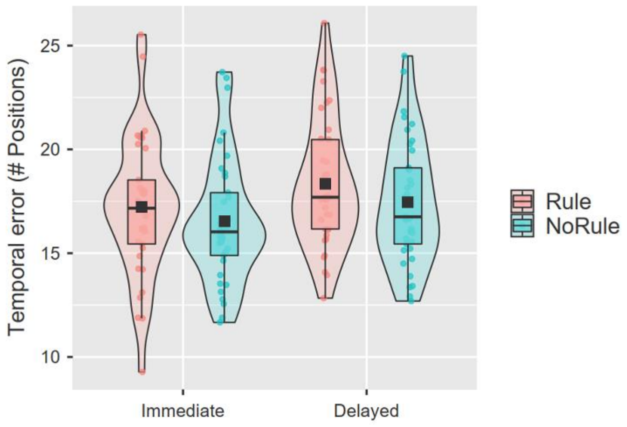

Experiment 2

(A) Face-location Memory

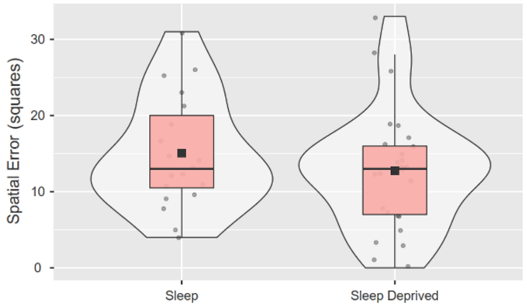

(B) Generalisation

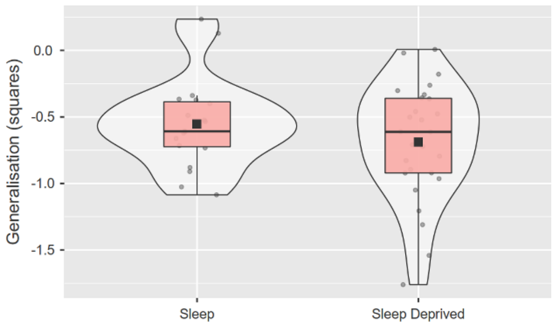

Results of Experiment1 (first three panels) and Experiment 2 (second figure): The figures here represent the same data as shown in figures 5 and 6. We used a violin plot to illustrate the dispersion of data. Each dot represents a data point. The width in the density plot (shape of the violin) shows the frequency of values. The box shows the interquartile range. The horizontal line (whiskers) represents the 95% confidence interval. In the box plot, the horizontal line illustrates the median, and the filled square corresponds to the mean. The explanation of each panel is comprehensively given in the figure legends of Figures 5 and 6.

3 References

Fang, J., Rüther, N., Bellebaum, C., Wiskott, L., Cheng, S., 2018. The Interaction between Semantic Representation and Episodic Memory. Neural Computation 30, 293–332. https://doi.org/10.1162/neco_a_01044

Greve, A., van Rossum, M.C.W., Donaldson, D.I., 2007. Investigating the functional interaction between semantic and episodic memory: Convergent behavioral and electrophysiological evidence for the role of familiarity. NeuroImage 34, 801–814. https://doi.org/10.1016/j.neuroimage.2006.07.043

Menon, V., Boyett-Anderson, J.M., Schatzberg, A.F., Reiss, A.L., 2002. Relating semantic and episodic memory systems. Cognitive Brain Research 13, 261–265. https://doi.org/10.1016/S0926-6410(01)00120-3

Snowden, J.S., Griffiths, H.L., Neary, D., 1996. Semantic-Episodic Memory Interactions in Semantic Dementia: Implications for Retrograde Memory Function. Cognitive Neuropsychology 13, 1101–1139. https://doi.org/10.1080/026432996381674

Takashima, A., Bakker, I., van Hell, J.G., Janzen, G., McQueen, J.M., 2017. Interaction between episodic and semantic memory networks in the acquisition and consolidation of novel spoken words. Brain and Language, Sleep and language learning 167, 44–60. https://doi.org/10.1016/j.bandl.2016.05.009

Weidemann, C.T., Kragel, J.E., Lega, B.C., Worrell, G.A., Sperling, M.R., Sharan, A.D., Jobst, B.C., Khadjevand, F., Davis, K.A., Wanda, P.A., Kadel, A., Rizzuto, D.S., Kahana, M.J., 2019. Neural activity reveals interactions between episodic and semantic memory systems during retrieval. Journal of Experimental Psychology: General 148, 1–12. https://doi.org/10.1037/xge0000480
